# Inanimate Surfaces as a Source of Hospital Infections Caused by Fungi, Bacteria and Viruses with Particular Emphasis on SARS-CoV-2

**DOI:** 10.3390/ijerph19138121

**Published:** 2022-07-01

**Authors:** Agata Jabłońska-Trypuć, Marcin Makuła, Maria Włodarczyk-Makuła, Elżbieta Wołejko, Urszula Wydro, Lluis Serra-Majem, Józefa Wiater

**Affiliations:** 1 Department of Chemistry, Biology and Biotechnology, Faculty of Civil Engineering and Environmental Sciences, Bialystok University of Technology, Wiejska 45E Street, 15-351 Białystok, Poland; e.wolejko@pb.edu.pl (E.W.); u.wydro@pb.edu.pl (U.W.); 2 Faculty of Medical Sciences in Zabrze, Medical University of Silesia, Traugutta sq.2, 41-800 Zabrze, Poland; s68987@365.sum.edu.pl; 3 Faculty of Infrastructure and Environment, Częstochowa University of Technology, 69 Dabrowskiego Str., 42-201 Częstochowa, Poland; mwm@is.pcz.czest.pl; 4 Research Institute of Biomedical and Health Sciences, University of Las Palmas de Gran Canaria, 35001 Las Palmas de Gran Canaria, Spain; lluis.serra@ulpgc.es; 5 Department of Agri-Food Engineering and Environmental Management, Faculty of Civil Engineering and Environmental Sciences, Bialystok University of Technology, Wiejska 45E Street, 15-351 Białystok, Poland; j.wiater@pb.edu.pl

**Keywords:** nosocomial infections, inanimate surfaces, virus, bacteria, fungi, SARS-CoV-2

## Abstract

The carriers of nosocomial infections are the hands of medical personnel and inanimate surfaces. Both hands and surfaces may be contaminated as a result of contact with the patient, their body fluids, and touching contaminated surfaces in the patient’s surroundings. Visually clean inanimate surfaces are an important source of pathogens. Microorganisms have properties thanks to which they can survive in unfavorable conditions, from a few days to several months. Bacteria, viruses and fungi are able to transmit from inanimate surfaces to the skin of the patient and the medical staff. These pathogens include SARS-CoV-2, which can survive on various types of inanimate surfaces, being a potential source of infection. By following the recommendations related to washing and disinfecting hands and surfaces, and using appropriate washing and disinfecting agents with a broad biocidal spectrum, high material compatibility and the shortest duration of action, we contribute to breaking the chain of nosocomial infections.

## 1. Introduction

Healthcare-associated infections remain one of the leading causes of increased morbidity and mortality among patients. It is estimated that in the United States, about 1.7 million people develop nosocomial-related illness each year, and approximately 99,000 of them die [1]. The patient’s endogenous bacterial flora is considered to be the main source of nosocomial infections, however 20 to 40% of nosocomial infections are caused by cross-contamination, where the vector of pathogen transmission is the hands of medical personnel. Contamination of the hands of medical personnel may result from direct contact with the patient or be caused by touching infected surfaces in the patient’s environment. On the other hand, a patient can become infected by pathogens that cause nosocomial infections by coming into contact with contaminated surfaces in a medical facility [2,3,4]. All surfaces in healthcare facilities should be visibly clean, i.e., free of visible residues, e.g., body fluids. However, apparent purity does not always correlate with microbiological purity. Surfaces that are considered clean may be microbiologically contaminated and constitute a reservoir of infectious agents.

The risk of nosocomial infections is related to microbial contamination of the surface by Gram (−) bacteria, e.g., *Acinetobacter*, Gram (+), e.g., *Staphylococcus aureus*, viruses, such as corona-, noro- and rotaviruses, and fungi, e.g., *Candida*. Even a single contact of human skin with a contaminated surface can contribute to the transmission of the pathogen. The most easily transmitted diseases from inanimate surfaces to the skin are: *Escherichia coli*, *Salmonella* spp., *Staphylococcus aureus* (100% of cases), *Candida albicans* (90%), rhinoviruses (61%), HAV (33%) and rotaviruses (16%) [4,5,6,7]. Microorganisms on the hands can be transferred to various surfaces, from which they can re-infect other people, both patients and medical staff. Given the very low hand-washing compliance rate among healthcare professionals, the risks associated with contaminated surfaces cannot be overlooked.

Different countries have different rules for cleaning and disinfecting of surfaces, but surface disinfection always increases the level of microbiological cleanliness of the patient’s surroundings, thus preventing the occurrence of additional infectious complications, breaking the chain of infections and contributing to the prevention of infectious diseases. Therefore, there is a strong need to constantly improve the procedures of cleaning and disinfecting surfaces in healthcare facilities. Very high concentrations of some pathogens in body fluids, such as blood, which can remain on surfaces in the patient’s environment in very small amounts on the order of a few µL, pose a serious risk of infection. Therefore, disinfection procedures, not simply cleaning procedures, are important, because even the most effective washing does not completely remove microscopic residues of body fluids, which can be a carrier of infectious agents [8,9].

Currently, both due to their way of life, the specificity of work and epidemic threats, people spend most of their time indoors. Therefore, they are exposed to constant contact with potentially contaminated surfaces, both in workplaces and in hospitals, where, due to the epidemiological situation, a large number of people have stayed/are staying. Detailed knowledge of the survival of pathogens on inanimate surfaces is essential for the understanding and concomitant control of infectious diseases. Hygiene measures are an important element in infection prevention procedures, which reduce the risk of transmission of pathogens. Infection control to prevent hospital epidemics is mandatory and essential, especially in hospitals and other healthcare settings. Therefore, the occurrence and survival of various pathogens on various inanimate surfaces was analyzed in many experimental studies.

The purpose of this review is to provide a comprehensive summary of recent experimental data on the survival of various pathogenic microorganisms with a special emphasis on SARS-CoV-2, and to compile a review of the current scientific literature and evidence on the persistence of various nosocomial pathogens on inanimate surfaces. 

## 2. Methods

Literature research was carried out as follows: scientific papers written on the basis of experimental studies were found and analyzed in PubMed using a combination of the following search terms: (persistence OR survival) AND (inanimate surface) AND (bacteria OR virus OR fungi OR SARS-CoV-2 OR COVID 19), (maintenance OR survival) AND (surface OR disease) AND hospital. Additionally, “Species: humans” was used as a filter. Authors manually selected the publications used. Literature research was conducted between January and December 2021. Only human pathogens were selected and grouped into the following groups: enveloped viruses and non-enveloped, SARS-CoV-2, bacteria and fungi. Survival of pathogens was determined as follows: from each publication, data were separated and grouped according to the group of pathogens (non-enveloped and enveloped viruses; gram-positive, gram-negative and other bacteria; and fungi), area—type and material, temperature and relative humidity (RH).

## 3. Discussion

### 3.1. Factors Influencing the Persistence of Pathogens

In hospitals, viruses, bacteria and their spores as well as fungi are transmitted not only by infected patients, but also by hospital staff, visitors and inanimate hospital environments, including surfaces frequently touched by hands, called “high-touch surfaces”, which include, among others, door handles, light switches, surfaces in toilets and in the area where the patient is in the room [10]. The risk of nosocomial transmission depends on the ability of pathogens to persist on surfaces. The longer a microorganism can remain on an inanimate surface, the greater the risk of its transmission to the patient or hospital staff [11]. The ability of hospital pathogens to colonize and survive on inanimate surfaces depends on several factors, including relative humidity (RH), temperature, ability to form biofilm and properties of the material from which the surface is made, including its porosity and orientation (horizontal or vertical). Hand hygiene and personal protection against infections are likewise important. The characteristics of the microorganism itself are also crucial, including its ability to create spores (bacteria), cell structure and other individual characteristics for a given microorganism [10,12,13].

One of the main factors determining the survival of microorganisms in the hospital environment is relative humidity (RH). For most viruses, the ability to survive depends mainly on the presence of a lipid envelope, which increases its resistance to low RH [12]. In the case of bacteria, some Gram-negative strains (*Pseudomonas* sp., *Klebsiella* sp., *Enterobacter* sp.) prefer higher relative humidity and lower temperature. On the other hand, bacteria such as *E. coli*, some species of *Salmonella* sp. and *Proteus vulgaris* have a lower survival rate at an intermediate relative humidity (50–70%). Among Gram-positive bacteria, for example, *S. aureus* can survive at low relative humidity, while *Staphylococcus epidermidis*, *Bacillus subtilis* and *Streptococcus haemolyticus* have a lower survival at 50–70% humidity. The different survival rate of bacteria depending on the humidity depends mainly on the structure of the cell wall. Consequently, Gram-positive bacteria, unlike Gram-negative bacteria, are more protected from physical stress and need less moisture to survive. In the case of fungi, it is argued that they prefer a moist environment [10].

Temperature is another important factor determining the survival of nosocomial pathogens. As reported by Kim et al. and Williams et al., lower temperatures result in longer survival times [14,15]. Often, the survival of microorganisms at low temperature depends on humidity, which can reduce drying stress [16]. In addition, high temperatures may reduce the activity of enzymes or damage the protein envelope of both the viral genome (RNA or DNA) and bacterial cells [4]. 

The factor that undoubtedly causes the persistence of microorganisms on inanimate surfaces is the ability to form a biofilm. It is the dominant life form of microorganisms in an ecosystem rich in nutrients [11]. The growth of pathogens in the biofilm makes them resistant to disinfection, which is associated with an increased production of extracellular substances such as proteins and polysaccharides after attachment to the surface. Biofilms maintain humidity and nutrients, and at the same time protect microorganisms against unfavorable physical and chemical factors, including disinfection. In a hospital environment, biofilm can be created on a variety of surfaces and objects such as blind cords, rubber sink stoppers or plastic parts of doors, windows, tables [17].

The survival of microorganisms in hospital conditions also depends on the type of surface and its characteristics. Polyvinyl chloride, ceramic tiles, and stainless steel are typical surfaces found in hospitals. Their features, such as porosity, hydrophobicity and free energy of the surface, determine the adhesion of microorganisms and the possibility of creating a biofilm [18,19,20]. As reported by Lagha et al., stainless steel is a material that promotes biofilm formation and can promote cross-contamination. Other materials such as ceramics or PVC can also be a source of disease transmitted from inanimate surfaces [21].

Pathogens with specific microbiological properties take part in the contamination of hospital environments. Such microorganisms must be able to survive long periods on an inanimate surface and to maintain virulence after exposure to environmental agents. In addition to the ability to contaminate surfaces in a hospital environment, they should also be able to easily colonize patients and temporarily colonize the hands of medical workers, which are the main vector of infection. Moreover, such microorganisms are infectious even in low doses and show relative resistance to active substances commonly used in surface disinfection preparations [22,23]. As mentioned earlier, survival on inanimate surfaces may be determined by the structure of the cell (in the case of Gram-negative and Gram-positive bacteria) or the presence of a lipid envelope (viruses). Other interesting features of microorganisms determining their persistence include increased survival of *Haemophilus influenzae* due to the activity of urease at low pH, or the presence of protein A, responsible for virulence and immunogenicity in *Staphylococcus aureus* [24,25].

### 3.2. Viruses

Viral infections can cause very different clinical courses, ranging from asymptomatic infections to severe, potentially fatal diseases. Viruses have the ability to contaminate and survive on inanimate surfaces. More than forty years ago, the potential role of inanimate surfaces in the transmission of certain viruses was highlighted by examining their ability to persist on several types of surfaces [26]. Hygiene and surface disinfection are particularly important when dealing with influenza, parainfluenza, intestinal viruses, hepatitis B and the coronaviruses causing SARS. It is believed that the SARS-causing coronaviruses are sprayed mainly by droplets; however, oral-fecal transmission and transmission via contaminated surfaces cannot be ruled out. These viruses can survive 24–72 h on plastic-laminated surfaces [27]. According to Sizun et al., human coronaviruses may survive on aluminum, cotton and latex a few h after drying, which could be linked with the possibility of person-to-person transmission through hand contamination from inanimate surfaces [28]. This was also confirmed by Kramer (2006), who pointed out that a significant number of viruses strains infecting the respiratory tract, such as coronaviruses, influenza or coxsackie viruses, consist of a potential source of transmission existing on surfaces even for a few days [4]. 

The survival of selected viruses (surrogate coronaviruses, gastroenteritis virus (TGEV) and mouse hepatitis virus (MHV)) on hard, non-porous surfaces under various conditions of air temperature (AT) and relative humidity (RH) was also investigated. TGEV and MHV can survive for up to several days on surfaces in AT and RH typical of healthcare environments. Generally, the genomes of RNA viruses are more sensitive to temperature than DNA viruses, but high temperatures can also affect DNA integrity. For some types of viruses, such as polioviruses and adenoviruses, low temperature increases their durability in a hospital environment [4]. Most respiratory viruses (e.g., influenza virus, coronavirus), herpes virus, rubella, measles and others that have a lipid envelope have the ability to survive in an environment with a lower relative humidity, ranging from 20 to 30%. In turn, viruses without a lipid envelope (enteroviruses, adenoviruses, rhinoviruses) can survive at a higher RH of 70–80% [12]. This means that enveloped viruses remain infectious on surfaces long enough to pose a risk of exposure, and lead to infection and disease transmission [29].

**Figure 1:**
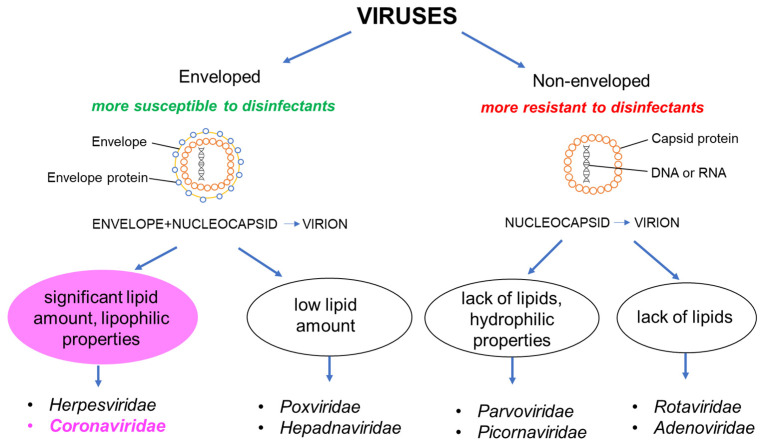
Virus classification based on structure.

Viruses can be classified according to their structure, which also determines their susceptibility to chemical disinfectants. Viruses consist of two components: the viral genome (RNA or DNA) and the virus-encoded protein capsid surrounding the genome. If a virus contains these two elements, it is called a non-enveloped virus. If a virus particle contains an additional external lipid bilayer membrane surrounding the protein capsid, it is called an enveloped virus. Non-enveloped viruses such as polio virus, hepatitis A virus (HAV) and parvovirus are characterized by strong hydrophilic properties and, subsequently, the highest resistance to disinfectants. On the other hand, viruses with reduced hydrophilic properties, such as rotaviruses, noroviruses and adenoviruses, are a little more susceptible to disinfection. The sensitivity of enveloped viruses to disinfectants depends on their lipid content. Those with low lipid content are more resistant than those with high lipid content. This is due to the fact that the lipid envelope is easily destroyed by chemical compounds that affect lipids such as alcohol, ether, chlohexidine, etc. The group of viruses most sensitive to chemical disinfectants includes enveloped viruses such as HIV, herpes virus, HCV hepatitis, and, importantly in the context of the pandemic, coronaviruses (Figure 1) [30,31].

**Figure 2:**
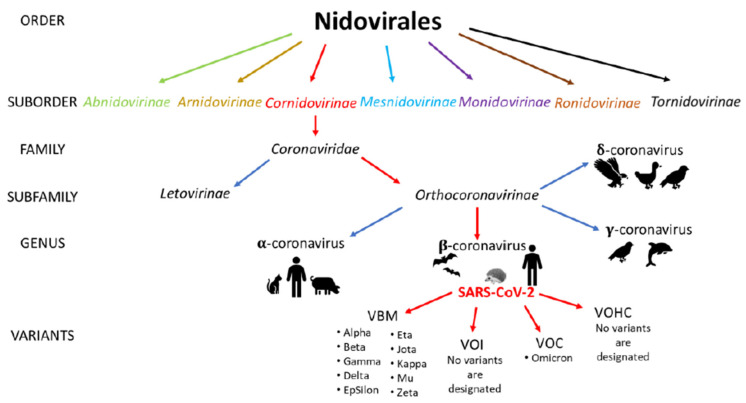
Virus classification (VBM—Variants Being Monitored; VOI—Variant of Interest; VOC—Variant of Concern; VOHC—Variant of High Consequence).

SARS-CoV-2 is one of the viruses from the family Coronaviridae and order Nidovirales. The family Coronaviridae is divided into two subfamilies, Letovirinae and Orthocoronavirinae, and SARS-CoV-2 belongs to the latter (Figure 2) [32]. According to the CDC, only Omicron was selected as a SARS-CoV-2 Variant of Concern, and no variants were designated as variants of interest or variants of high consequences. In April 2022, the Delta variant was downgraded from a Variant of Concern to a Variant Being Monitored because of evidence indicating that it does not currently pose a significant risk to public health in the United States [33].

**Figure 3:**
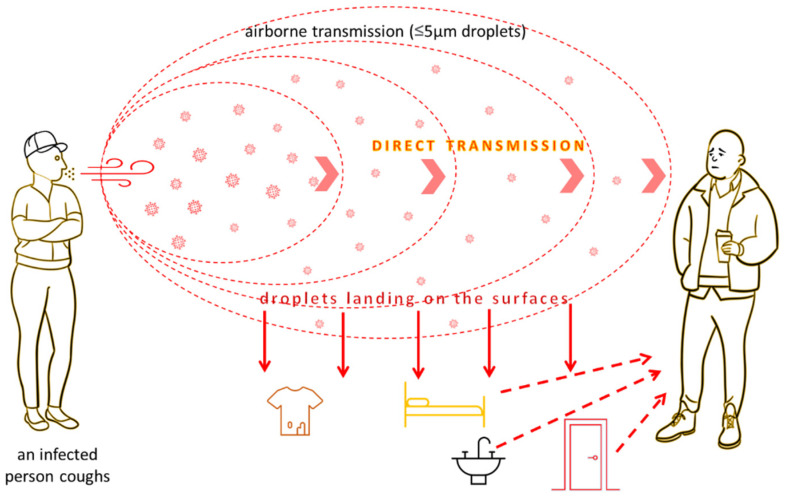
Potential routes of COVID-19 spread from coughing by an infected person.

By the end of 2019, only six different coronaviruses dangerous to humans were recorded, four of which resulted in mild symptoms of the common cold (HCoV-NL63, HCoV-229E, HCoV-OC43 and HKU1), while two have caused a pandemic in the past 20 years. The severe acute respiratory syndrome coronavirus (SARS-CoV) caused the SARS epidemic (10% mortality, 2002–2003). In contrast, the Middle East Respiratory Syndrome (MERS-CoV) coronavirus caused a pandemic in 2012 (37% mortality) [34]. All viruses from the group of coronaviruses that turned out to be pathogenic to humans were characterized by zoonotic origin. Coronaviruses are positive-strand RNA viruses found in many species of animals, often showing no disease symptoms in their hosts. On the basis of genetic and serological characteristics, four types of them have been distinguished: alphacoronavirus (alpha-CoV), betacoronavirus (beta-CoV), gamma-coronavirus (gamma-CoV) and deltacoronavirus (delta-CoV) (Figure 2). Gammacoronaviruses and deltacoronaviruses are mainly found in birds, although gammacoronaviruses also infect some cetaceans, including beluga whales and bottlenose dolphins. In contrast, alphacoronaviruses and betacoronaviruses occur mainly in mammals. SARS-CoV and MERS-CoV have spread to humans from civet cats and dromedary camels [35]. The closest known relative strain of SARS-CoV-2 is the coronavirus strain RaTG13 found in a bat *Rhinolophus affinis* from Yunnan Province, China in 2013. The degree of similarity at 96% may suggest a relatively close relationship between the two virus strains [36]. One of the possibilities is that the SARS-CoV-2 ancestor has been incubating for years inside bats, accumulating mutations, and probably through a random event, the virus was transmitted in humans. It should be also emphasized that both civets and racoon dogs, which are susceptible to SARS-CoV-2 infections, were sold live in Wuhan in 2019, and a market in Wuhan was an epicenter of SARS-CoV-2 infection [37]. SARS-CoV-2 turned out to be much more contagious than other viruses in the family, as evidenced by its extremely rapid spread in over 180 countries around the world. This feature, combined with the often severe course of the infection, has raised concerns about the possible breakdown of healthcare systems that will not be able to treat and save a large number of cases at the same time. [38]. Therefore, both governments and public health sectors are trying to contain this pandemic and avoid a catastrophic scenario. As there are no effective and safe antiviral drugs for SARS-CoV-2, infection control is currently the only available method, apart from vaccination, to limit the spread of the virus. The choice of preventive measures to control infection depends largely on knowing and understanding the routes of transmission of the virus. COVID-19-related pneumonia diagnosed in both hospital and family settings provided the basis for describing the direct route of person-to-person transmission of the virus [39,40]. However, indirect transmission pathways, such as fecal-oral, hospital, airborne, and contact with contaminated surfaces, are also believed to play an important role (Figure 3) [41]. 

**Figure 4:**
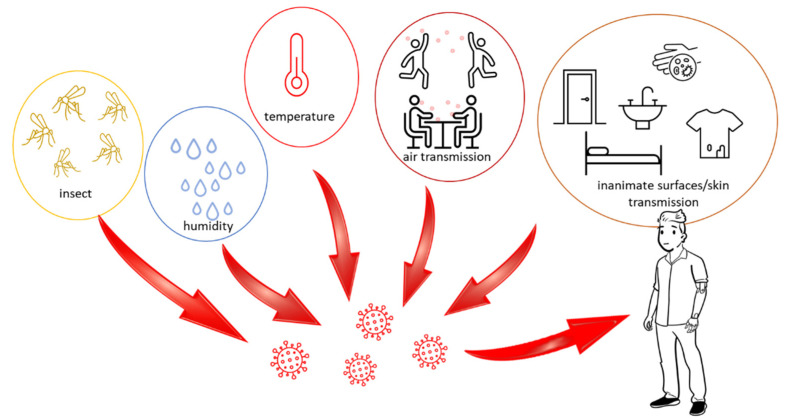
Environmental factors that play a potential role in the spread of SARS-CoV-2: Temperature, humidity, insects, air transmission, inanimate surfaces and human skin.

The dependence of the stability and rate of spread of the virus on environmental conditions such as temperature, relative and absolute humidity and sunlight is poorly understood. Coronaviruses, due to the fact that they are enveloped viruses and are more sensitive to heat, are able to survive longer in lower temperatures and relative humidity [42]. The study of these dependencies is necessary to be able to construct a robust and long-term protocol to interrupt the indirect transmission of SARS-CoV-2 to the environment, limit its spread and thus minimize the risk. Research has also been carried out on the potential for transmission of SARS-CoV-2 by house flies (*Musca domestica*). Flies generally can transmit microbes from waste-contaminated breeding habitats to food consumed by humans. Although flies theoretically can transfer SARS-CoV-2 virus mechanically, the results of the research indicate that flies do not play a significant role in the transmission of SARS-CoV-2 to humans and animals [43]. Mosquitoes also do not play a fundamental role in the transmission of SARS-CoV-2 to humans (Figure 4) [44,45]. 

The ability of a given virus to survive in an inanimate environment is a prerequisite for its spread. Important factors determining the level of infectivity and the extent and speed of spreading a virus are its characteristics, but also the biotic and abiotic properties of the environmental surface it contaminates, as well as environmental conditions. Therefore, SARS-CoV-2 is considered to persist for a long time on environmental surfaces. The adhesion mechanism used by the SARS-CoV-2 virus on various types of inanimate surfaces has not yet been fully elucidated. However, electrostatic interactions, hydrophobic effects and, to a lesser extent, non-covalent bonds such as van der Waals forces are believed to play a large role. All these factors can influence the adhesion of the S protein to the surface [46,47]. Literature data indicate that the load on the surface of the virus varies with the pH of the environment. Viral particles are characterized by high stability in a wide range of pH, however, they are more stable at alkaline pH. Protein E, which is part of the hydrophobic layer surrounding the viral particle, also plays an important role in viral adhesion [48,49].

SARS-CoV-2 virus RNA has been detected on the surface of door handles, cell phones, and other items in the homes of confirmed cases. To date, limited data are available on the survival of SARS-CoV-2 in the environment [50,51]. The durability of SARS-CoV-2 on surfaces made of plastic was tested in two experiments. In the first study, SARS-CoV-2 maintained its infectivity for 4 days and completely degraded after 7 days on a plastic surface at room temperature and 65% relative humidity. The second study showed that SARS-CoV-2 maintained its infectivity for 3 days on the plastic surface at room temperature. There was also no difference between the persistence of SARS-CoV-2 and SARS-CoV-1 on plastic surfaces, and both viruses lost their infectivity completely after 4 days. Literature data show that the survival rate of coronaviruses on metal surfaces differs depending on the type of metal. They survive shorter on copper, nickel and brass surfaces than on stainless steel and zinc surfaces. SARS-CoV-2 was shown to persist on the surface of stainless steel for 3 days and only after 4 days did it become undetectable. This virus showed a lower survival on copper (4 h) compared to SARS-CoV-1 (8 h). This data was also confirmed by the study of Aboubakr et al. (2021), who concluded that the survival rate of SARS-CoV-1 and SARS-CoV-2 was significantly lower on copper, latex, and less porous fabrics than on surfaces such as metals (stainless steel and zinc), glass. Based on the obtained results, the authors suggested that the use of contact surfaces made of copper in hospitals may be a factor reducing the transmission of SARS-CoV-2. However, it should be noted that the coronavirus may have different survival rates on the same surface but under different temperature and relative humidity conditions [42]. The influence of temperature, relative humidity and droplet size on the stability of SARS-CoV-2 was tested in laboratory conditions on specially created matrices imaging non-porous surfaces. It was shown that the rate of decomposition of SARS-CoV-2 increased with increasing humidity or temperature, but the droplet volume (1–50 μL) and the type of surface (stainless steel, plastics or nitrile) did not have a significant effect on the parameter tested. Consequently, potential transmission can linger for hours to days in indoor areas, making it difficult to assess the risk of surface contamination [52].

The survival of SARS-CoV-2 on glass at room temperature and 65% relative humidity was also investigated. The virus remained contagious for 2 days and became completely undetectable after 4 days. The stability of SARS-CoV-2 drops significantly with increasing temperature and humidity. Values above 38 °C and 95% cause the adhesion of virus particles to the surface to be much weaker. Sunlight also acts as a natural virus inactivating agent on surfaces [48,53]. Of the swab samples taken from inanimate surfaces in the infectious disease emergency department, only two were positive for low SARS-CoV-2 levels, and none caused a cytopathic effect on day 7 of the study. Therefore, it is believed that contact with inanimate surfaces in contaminated areas may indicate the possibility of infection, but not as extensively as is believed [54].

The survival rate of SARS-CoV-2 on cardboard compared to SARS-CoV-1 was also analyzed. SARS-CoV-2 survived longer (1 day) than SARS-CoV-1, which only survived 8 h under the same conditions. Comparing surfaces with different porosity, it was shown that SARS-CoV-2 survived longer (days) on surfaces with greater porosity than those with lower porosity (hours). Virus survival times on the inner and outer layers of the surgical masks were 4 and 7 days, respectively, and 2 days on fabric and 1 day on banknotes. On paper, the virus only survived 30 min with a total degradation time of 3 h [48,55]. Studies conducted and published by Kampf et al., van Doremalen et al., Ren et al., and Rawlinson et al., confirmed that SARS-CoV-2, similarly to other coronaviruses, could persist on inanimate surfaces such as plastic, glass and metals for a few days: from 72 h to even 9 days. However, they also pointed out that SARS-CoV-2 as an enveloped virus is susceptible to surface disinfection procedures conducted with the use of most cleaning agents [55,56,57,58]. 

Influenza virus is also an infectious agent in the environment even after thorough drying of the surfaces on which it is located, and it can be re-sprayed as an aerosol when cleaning floors. It can survive 24–48 h on non-porous surfaces and be reapplied to the skin, causing staff–patient and patient–staff cross-contamination. Parainfluenza virus exhibits similar resistance to desiccation and can survive up to 10 h on non-porous surfaces and up to 6 h on clothes [59]. A 2016 study found that influenza A (H1N1) viruses can persist and remain infectious on stainless steel surfaces for 7 days [60]. 

Enteric viruses such as noroviruses belong to caliciviruses, single-stranded non-enveloped RNA viruses that are common pathogens in humans and animals. Viruses from the human norovirus group are the leading cause of epidemic and sporadic acute gastroenteritis (AGE) worldwide. Inanimate surfaces are considered to be the key carriers of the spread of human norovirus during the epidemic. It should also be mentioned that the literature data indicate that the virus easily spreads between inanimate surfaces and human skin [61,62]. Noroviruses cause extensive contamination of surfaces and it is possible to spread them in the form of aerosols when cleaning floors or other larger surfaces. They can survive on large surfaces for up to 12 days [63]. They can also be found on surfaces such as door handles, buttons in elevators, and toilet flushing cisterns. In the case of the presence of noroviruses in the environment, it is very important to disinfect the entire environment of the patient properly, because due to the long survival time of the virus and the small inoculum sufficient for infection, another outbreak may occur. In addition, these viruses are very resistant to chemical disinfection, so it is worth disinfecting contaminated surfaces with washing and disinfecting preparations several times, each time preparing a new solution of the preparation. Rotaviruses are very common food-borne gastroenteritis viruses, but hands and contact with contaminated surfaces are essential for the transmission of infection. These viruses are very infectious and remain on surfaces for a long time, up to 60 days [64,65,66].

HBV virus is very infectious and highly resistant to environmental factors. It does not lose infectivity for up to 6 months at room temperature, while at 60 °C it can survive up to 4 h. It is inactivated at 100 °C after 20 min and in an autoclave (121 °C) after 15 min. Due to the exceptional infectivity of the virus, surfaces should be considered as a possible source of HBV infection. The blood of an infected person is a high-risk factor, as 0.0001 mL of an infected person’s blood is enough to transmit an infection. Therefore, compliance with the rules of disinfection breaks the chain of infection to some extent [67,68,69].

### 3.3. Bacteria

In the hospital environment, the most common bacterial strains causing difficult-to-control diseases include: *Clostridium difficile*, *Klebsiella pneumoniae*, *Acinetobacter species*, *Escherichia coli*, *Enterobacter sakazakii*, *Enterobacter cloacae*, *Pseudomonas aeruginosa* and *Staphylococcus aureus*, subdivided into two groups (MRSA-methicillin-resistant *Staphylococcus aureus* and VRSA-vancomycin-resistant *Staphylococcus aureus*). 

Katzenberger et al. described the percentage share of bacteria which play the main roles in outbreak events in hospitals, e.g., *S. aureus* (11.9%), *K. pneumoniae* (7.9%), *P. aeruginosa* (7.1%), *A. baumannii* (7.0%), *S. marcescens* (4.6%), *E. faecium* (3.6%), *E. coli* (2.4%) and *E. cloacae* (2.3%) [70]. According to Kramer et al., single-handed contact with a contaminated surface will have a variable degree of transmission depending on the bacteria. It was observed that transmission from contaminated surfaces to hands was most effective in the case of *S. aureus* MRSA or VRE, *E. coli* and *Salmonella* spp. [71]. The multiresistance of bacteria to antibiotics is a serious problem not only in terms of their prolonged persistence on surfaces, but also in limiting the possibilities of appropriate treatment. The longevity of bacteria increases the risk of spreading them in the hospital environment, especially in the conditions that exist in routine care of patients [72]. According to Katzenberger et al., 2021, the most susceptible bacteria to spread in the hospital environment are strains that are resistant to antibiotics, because they can more easily form biofilms on various surfaces, which may increase the likelihood of prolonged life and facilitate further spread [70]. 

It is widely recognized that these pathogens are not able to be completely eliminated from the hospital environment, but they can be reduced to a minimum. In order to decrease their prevalence, an in-depth knowledge of the resistance mechanisms of these pathogens is essential, as is a precise definition of universal principles of infection prevention, hygiene practice and a correct antibiotic policy.

*Clostridium difficile* is an anaerobic, Gram (+), spore-forming and toxin-producing rod. It is part of the physiological human intestinal flora in almost 3% of the healthy population and in 20–30% of those hospitalized. It exists in a vegetative form of spores in the intestine or outside of it. The vegetative form can survive on a dry surface at room temperature for up to 15 min, and on a wet surface for up to 6 h. *Clostridium difficile* spores are characterized by high resistance to alcohol-based disinfectants and other chemicals used in hospitals, UV radiation, high temperatures, etc. (Table 1). 

The high resistance results from the stratified structure of the spore. The core inside contains dehydrated cytosol, DNA, RNA and enzymes. The core is covered with an inner membrane, a wall, and then a cortical layer (a modified peptidoglycan that will later be involved in the formation of the cell wall of the vegetative form). Outside, it is covered with a mantle containing mainly proteins, dipicolinic acid and calcium ions, which form calcium dipicolinate. Therefore, *Clostridium difficile* spores are very tough and resistant to traditional surface cleaning methods [73]. *C. difficile* spores have a strong ability to adhere to a variety of surfaces, including plastic laboratory equipment. They can survive on the surfaces of hospital equipment for up to 5 weeks, and on hospital floors for up to 5 months. Literature data show that the storage of spore-contaminated materials at 4 °C and the freezing and re-thawing cycles also did not reduce their number [94,95].

Contamination of the inanimate environment by *C. difficile* has been reported in close proximity to an infected patient. The contamination rate was up to 58%, and beds, cabinets, pressure measuring devices, walls, floors, washbasins and furniture were particularly frequently infected. Hospital floors can remain infected with *C. difficile* for up to 5 months, and the rate of contamination increases in the presence of colonized patients or those patients with diarrhea [96]. Molecular techniques provide compelling evidence of *C. difficile* transmission from environmental surfaces to the patient. According to the data obtained with their help, it was found that surfaces in the patient’s environment serve as a reservoir of the germ and allow cross-colonization of patients after their contact with healthy healthcare professionals [97,98]. Among the hospital surfaces where *Clostridium difficile* has been found, in addition to the walls and floors, there are also cabinets, beds, swimming pools, blood pressure cuffs, sinks, and occasionally shoes and stethoscopes [99,100].

*Staphylococci*, which are microorganisms that easily contaminate the hospital environment, can be divided into two groups: coagulase-positive, e.g., *Staphylococcus aureus*, and coagulase-negative, e.g., *Staphylococcus epidermis* and *Staphylococcus saprophyticus*. *Staphylococcus aureus* is especially common in healthcare professionals, diabetics and people with venous catheters. However, the greatest threat is MRSA, or methicillin-resistant *Staphylococcus aureus*, which causes nosocomial infections characterized by particularly high morbidity and mortality rates [101]. *Staphylococcus aureus* was one of the most common pathogens associated with nosocomial infections reported to the NHSN (National Healthcare Safety Network) from January 2006 to October 2007. MRSA constitutes 56% of all *S. aureus* isolates from hospital equipment and supplies. The most important reservoir of MRSA in a hospital is colonized or infected patients, who easily contaminate medical and electronic equipment in their environment. MRSA can survive on dry surfaces for up to several months [102,103,104]. Although the primary mode of MRSA transmission to patients is through the colonized hands of healthcare professionals, there is evidence that exposure to MRSA contaminated surfaces may also cause patient infections [105,106]. Literature data indicate that *S. aureus* remains viable on dry surfaces for periods from 1 week up to even 3 years [90]. While there are data showing the survival of *S. aureus* in home and healthcare settings, little attention has been paid to the spread of these organisms, including MRSA, across the community [107]. Table 1 shows quite varied survival of *S. aureus* on various inanimate surfaces, e.g., polyethylene for 90 days, sterile packages for 266 days, screw cap bottles for 318 days and polypropylene for over 1097 days [77,89,108,109]. It seems, however, that these findings are the outcomes of the optimization of the experimental conditions. They used staphylococcal strains with a high level of resistance to desiccation and an inoculum of 10^7^–10^9^ CFU. These results were clear and consistent under laboratory conditions, but they may not accurately reflect *S. aureus* survival in the community. Domon H. demonstrated that hazardous and pathogenic *S. aureus* strains, including MRSA, which cause healthcare-associated infections, are rarely isolated from inanimate surfaces in the community, because of their poor survival on a dry surface for more than 24 h [110].

*Acinetobacter* is a group of Gram (−) bacteria that do not ferment glucose. It includes 32 species, of which *Acinetobacter baumannii* is the most important, constituting up to 70% of isolates. In recent years, an increase in *Acinetobacter* resistance and an increasing number of nosocomial infections caused by these microorganisms have been observed. This is due to their ability to survive in the environment both on dry and wet surfaces for a long period of time (weeks) in a wide range of temperatures and environmental pH. In vitro studies showed that *Acinetobacter* can survive on ceramic surfaces, stainless steel, rubber and polyvinyl chloride, and relatively higher humidity promotes bacterial growth [111,112,113]. Therefore, in a hospital environment, *Acinetobacter* can most often be found in humid places such as bathrooms, mops, respirators and air humidifiers. These strains have the ability to produce a biofilm that hinders the penetration of disinfectants and increases bacterial resistance to decontamination.

The microorganisms of the genus *Enterococcus* colonizing the human gastrointestinal tract belong to the physiological flora, and are actually resistant to many antibiotics, e.g., penicillin, aminoglycosides and glycopeptides. There are 43 species here, but the most common are *Enterococcus faecalis* and *Enterococcus faecium*. Vancomycin-resistant enterococcal (VRE) strains were first isolated in 1986. Currently, they account for 1/3 of all nosocomial infections caused by contamination of the hospital environment, especially surfaces and medical equipment. VRE causes serious infections in immunocompromised patients. Transmission of VRE in hospitals is usually associated with transient colonization of the hands of medical personnel. However, there are literature reports showing the role of hospital surfaces and medical equipment as vectors for VRE [114]. Katzenberger et al. studied the survival kinetics of the selected microorganisms on four different types of surfaces, such as polyvinyl chloride, glass, stainless steel and aluminum (Table 1). The scholar stated that *A. baumannii* and *E. faecium* showed the highest survival capability regardless of the material of the surface. Viable bacteria of those two species remained detectable even at the end of the entire observation period of one month [70]. Transmission of both *A. baumannii* and *E. faecium* through contaminated surfaces can easily occur if appropriate preventive infection control measures are not taken. These pathogens, being able to survive for several days on inanimate surfaces, pose a significant risk of transmission, e.g., in hospitals. Thus, in the event of high disease rates, qualified personnel should carefully search for previously unidentified areas or violations of standard decontamination procedures if the spread of the pathogen continues despite extensive cleaning and disinfecting efforts.

Many studies confirm the presence of *Pseudomonas aeruginosa* in sinks and drains in plumbing, but it is still unclear whether using sinks leads to an increased risk of the spread of this microorganism. *P. aeruginosa* isolates from inanimate surfaces were not always compatible with patient isolates. Most infections tend to be caused by the patient’s endogenous flora, but surfaces and medical equipment as possible sources of infection cannot be ruled out [115,116]. The literature data presenting experiments regarding survival of different bacterial species on various surfaces, like glass, stainless steel, polyvinyl chloride, and aluminum (Table 1), indicate that *P. aeruginosa* was completely inactivated in less than two days [70]. Neely AM presented the results regarding the survival rate of clinical and environmental strains of *P. aeruginosa* survival rate on different textiles such as cotton, polyester and polyethylene. It amounted to 2 h–7 days when inoculum was 10^4^–10^5^ CFU [77]. 

### 3.4. Fungi

Since the 1980s, there has been a continuous increase in the number of fungal infections, also proving that fungi have become a common pathogen causing hospital infections. Therefore, there is a need to evaluate the mycological flora in studies involving personnel, patients, walls, floors and equipment.

Nosocomial fungi have a wide range of temperature (0–60 °C) and pH (2–8.5) tolerances and grow more effectively on wet surfaces. *Candida* fungi are primarily isolated from hospital surfaces and can survive for up to 4 months. Although the vast majority of infections caused by *Candida* result from endogenous sources, molecular studies of yeast obtained from patients collected by medical personnel and from the medical community proved the transmission of *Candida albicans*, *Candida glabrata* and *Candida parapsilosis* among patients [117,118]. 

*Candida* infections are a serious problem in hospitals due to high patient mortality, especially in patients with low immunity, as well as long-term and costly treatment. Systemic infections caused by *Candida* are the fourth leading cause of nosocomial bloodstream infections. Candidiasis caused by *Candida* sp. is mainly the result of long-term antibiotic therapy with a broad spectrum of activity. In addition, one of the features that causes *Candida* infection and the possibility of its transmission between hospital patients is the ability to form biofilm, which at the same time makes it difficult to combat, both by drugs and by disinfecting the hospital space. In recent years, in hospitals in many countries, special attention has been paid to the strain *C. auris*, which is characterized by multi-drug resistance and is difficult to control due to its long-term survival ability on both wet and dry surfaces [119]. According to Shield et al. and Schelenz et al., the causes of *Candida* infections in hospitals are, apart from the hands of healthcare workers, hospital surfaces and medical tools and equipment [120,121]

The surfaces can be intensively and permanently contaminated by fungi of various species, as shown by experimental inoculation of dry surfaces with *C. albicans* (3 days) and *C. parapsilosis* (14 days) [122]. Molecular typing of *Candida* isolates obtained from the hospital environment and from patients proves that the endemic species of *C. albicans* and *C. glabrata* constitute the environmental reservoir of *Candida*. *Candida* strains obtained from patients were identical to those found on hospital surfaces [123,124]. According to Traore O et al., *Candida parapsilosis* survives much better than *C. albicans* on non-porous surfaces such as glass and stainless steel. *C. albicans* was undetectable at the end of the third day of the experiment, while *C. parapsilosis* was detectable even after 14 days of incubation under ambient conditions. *C. albicans* survived also on textiles (polyester + cotton and 100% cotton) significantly better than on glass and metal, while *C. parapsilosis* survived on both fabrics as well as on non-porous supports. Colony forming units of both *Candida* species remained detectable even after 14 days of storage under ambient conditions [122].

Both the reservoirs and the methods of transmission of *Aspergillus* and *Zygomycetes* are similar. In hospitals, the sources of *Aspergillus* are contaminated air filtering systems, ventilation systems contaminated with dust accumulated during renovation or construction, carpets, food and plants [125,126]. The species of the genus *Aspergillus* most commonly causing nosocomial infections are *A. fumigatus*, *A. flavus* and *A. terreus*. They are filamentous fungi which are characterized by a great variety and the ability to colonize various habitats around the world. They can cause a disease called aspergillosis, which presents with non-invasive infections of the respiratory tract, ears, or eyes. Invasive *Aspergillus* sp. infections may occur after immunosuppression or surgery, which may even result in death [126]. Research by Neely A. and Orloff M. indicates plastics and materials commonly used in hospitals as important reservoirs and vectors for the transmission of fungal infections. The tested species of fungi were able to survive on inanimate surfaces for at least one day, and very often for several weeks [127]. The method recommended for preventing fungal nosocomial infections is constant monitoring of air pollution, but so far there is no standardization of the methodology used to identify fungal contamination. As traditional methodologies are characterized by low precision and overly-long measurement time, flow cytometry is recommended as a method that can be validated for a specific measurement [128]. The most effective method of fighting fungal nosocomial infections is the constant involvement of people working in healthcare facilities in widely understood preventive measures, removing potential causes of infections and monitoring the possibility of pathogens in the patient’s environment. According to Baudisch et al., Mold fungi are commonly present, resistant to temperature and easily adsorbed on dust particles, which makes them durable for up to several months [22,129,130] 

### 3.5. Hygiene and Disinfection of Surfaces

**Figure 5:**
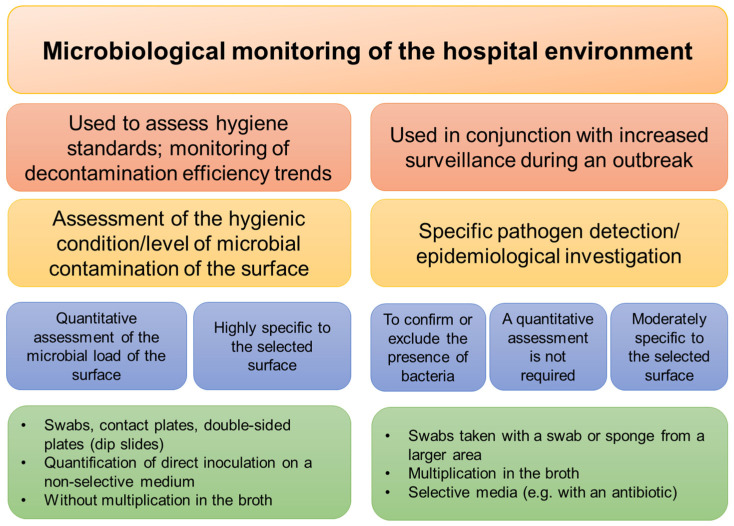
Microbiological monitoring of the hospital environment.

Contaminated surfaces and medical equipment can clearly contribute to the spread of nosocomial infections by infecting the hands of healthcare professionals and, indirectly, patients, or they can infect patients directly. Therefore, both an implementation of microbiological monitoring of the hospital environment and cleaning/disinfection of surfaces is essential to prevent cross-contamination (Figure 5). 

**Figure 6:**
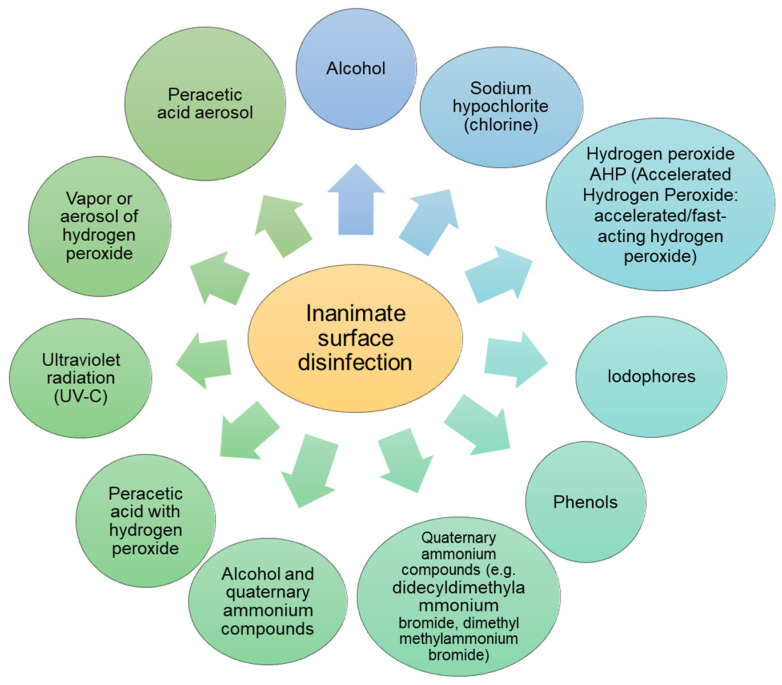
Inanimate surface disinfection.

Cleaning and disinfection recommendations include, among others, implementing protocols based on empirically proven guidelines for cleaning and disinfecting hospital surfaces and medical equipment [131]. The second recommendation is cleaning and disinfecting of surfaces that are easily and frequently contaminated during routine activities, especially frequently touched surfaces, such as: beds, bedside cabinets, door handles, bathroom fittings in patients’ rooms and equipment in the patient’s immediate vicinity. For the disinfection of small surfaces, preparations based on hydrogen peroxide, most often in sprays, are recommended, which are easy to use and, if they show sporicidal activity, can also be used in the environment of a patient infected with *C. difficile.* Since *C. difficile* spores are resistant to alcohol and various commonly used disinfectants, in the case of *C. difficile* infection, the use of chlorine-based disinfectants on medical equipment in close proximity to a patient infected with *C. difficile* is recommended. Due to their antimicrobial activity, preparations containing per-acetic acid and hydrogen peroxide are also recommended [132]. In cases of possible SARS-CoV-2 contamination, the WHO recommends cleaning surfaces frequently with water, detergents and disinfectants. Environmental cleaning and disinfection procedures should be conducted consistently and correctly with the use of ethanol at >70% concentration, povidone iodine, sodium hypochlorite and quaternary ammonium compounds with alcohol [56,133,134]. (Figure 6). One of the innovative methods of surface disinfection is the use of the non-thermal plasma sterilization technique, also known as “cold plasma”. The following technique types can be distinguished: direct current (DC) corona discharge, atmospheric pressure plasma jet (APPJ) microwave, dielectric barrier discharge (DBD), micro-hollow cathode discharge (MHCD) jet and pin-to-hole spark discharge (PHD) plasma. Non-thermal plasma techniques can be applied to medical devices without noticeably affecting their structure [135]. The action of plasma on microorganisms is based on the denaturation of proteins, inactivation of enzymes and DNA mutation. For example, treatment of *E. coli* with an atmospheric pressure plasma jet (APPJ) can cause cell-membrane damage and consequent cell lysis [84]. In the case of *S. aureus*, the use of plasma as a pre-sterilization step increases the susceptibility of *Staphylococcus aureus* to antibiotics [136]. Studies were also carried out on the effect of plasma on the destruction of enveloped and non-enveloped viruses, in which the inactivation of, e.g., influenza and RSV (enveloped viruses) and adenovirus (non-enveloped) [135]. The use of plasma seems to be a future tool in surface disinfection, but the influence of plasma on biomacromolecules (cell membranes, cell walls or membrane proteins and polysaccharides) or the mechanism of mutation and cell death is still unexplained and is the subject of research.

**Figure 7:**
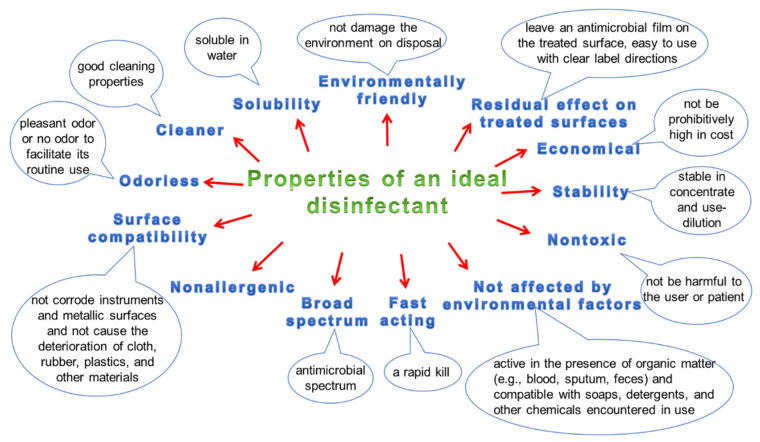
An ideal disinfectant.

On the other hand, noroviruses are resistant to the activity of many commonly used disinfectants; therefore, a preparation based on chlorine compounds or another preparation in the spectrum which also includes noroviruses should be used (CDC). Properties of an ideal disinfectant are presented in Figure 7. When choosing a disinfectant, one should take into account both the type of disinfected surface and the degree and type of its contamination. A very important aspect is also the possibly ecological, environmentally friendly formula of the preparation and its possibly non-allergenic properties. The desirable properties of the biocide also include quick action, good water solubility (which allows for its easy preparation), as well as a broad spectrum of activity allowing the elimination of many types of pathogens at the same time. Considering surface disinfection, an additional positive aspect is the simultaneous cleaning and disinfecting effect of the preparation, which saves time and reduces disinfection costs, because it allows for the simultaneous conduct of two processes: washing and disinfection.

## 4. Conclusions

Scientific research has confirmed that contaminated hospital surfaces can be the cause of infection; therefore, cleaning and disinfection procedures should be carried out very carefully, selecting appropriate washing, disinfecting or washing-disinfecting agents with a broad biocidal spectrum and high efficiency, as well as in accordance with applicable standards and recommendations. Different microorganisms are characterized by different resistances to disinfectants, depending on the active substance used in the disinfectant. Bacterial spores (e.g., *Bacillus*, *Clostridium*) are generally the most difficult to combat with standard disinfectants. Their resistance due to their structure, which makes them resistant to disinfectants such as chlorhexidine, glutaraldehyde and QACs. The second most difficult microorganism to control with disinfection is *Mycobacteria*, followed by small non-enveloped viruses such as norovirus and poliovirus. Gram-negative bacteria are more resistant to disinfection than Gram-positive bacteria due to the presence of the cell wall composed of lipopolysaccharide and proteins in the former. Fungi and large non-enveloped viruses are more resistant to disinfection than lipid-enveloped viruses, which include SARS-CoV-2. Among all the above-mentioned microorganisms, SARS-CoV-2 belongs to the group that is most susceptible to removal from both inanimate surfaces and from the skin using traditional methods of washing and disinfecting. The above-mentioned literature data confirm the presence and the ability to survive on inanimate surfaces of the SARS-CoV-2 virus. Even in the first months of the global pandemic, its ability to travel through contact with contaminated surfaces was investigated as one of the possible contaminants. It turned out that it can be active and infectious on surfaces for a time range from hours to several days. However, because it is easily removable with commonly available surfactants and disinfectants, regular disinfection and proper hand washing can reduce viral transmission of both SARS-CoV-2 and other pathogens commonly found on inanimate surfaces in hospitals. Currently, one of the problems faced by hospitals is cross-transmission of pathogens, which at the same time poses a challenge in the search for methods of adequate control of surface contamination and the search for effective methods of its disinfection. The use of the correct disinfectant and following an effective cleaning procedure are key to preventing health and safety risks. A properly selected preparation for a specific surface and the degree of its contamination ensures that the microorganisms will be completely removed, not spread accidentally, and any additional threats will be minimized. 

## Figures and Tables

**Table 1:** Selected bacterial strain survival on inanimate surfaces and their resistance to disinfectants.

Pathogen	Material	Survival on Surfaces	Susceptibility/Resistance to Disinfectants	Physical Inactivation	Ref.
*Clostridium difficile*	Stainless steel	>6 weeks	Clostridium spores are resistant to ethyl and propyl alcohols; high level disinfectants such as 2% glutaraldehyde, 8% formaldehyde and 20 ppm sodium hypochlorite can kill spores within 20 min	inactivated by moist heat at 121 °C for 15–30 min	[73,74,75]
	Glass	15 min			
	Flooring material	5 months			
*Klebsiella pneumoniae*	Plastics surfaces	9–32 days	Gram-negative bacteria are susceptible to disinfectants including phenolic compounds, alcohols (70% ethanol), hypochlorites (1% sodium hypochlorite), glutaraldehyde, iodines (0.075 g/L) and formaldehyde (18.5 g/L; 5% formalin in water).	Reduction in the growth and metabolic activity at temperatures > 35 °C and significant growth reduction at 60 °C;	[76,77,78,79]
	Stainless steel	3–6 weeks			
	Ceramics/Flooring material	2 weeks			
	fabrics	<1 h–4 weeks			
*Acinetobacter* sp.	Glass	7–20 days	Susceptible to disinfectants such as povidone-iodine, 0.5% chlorhexidine digluconate, 70% ethyl alcohol and didecyl dimethyl ammonium chloride in combination with N-(3-aminopropyl)-N-dodecylpropane-1, 3-diamine; glutaraldehyde-based product has a high-level disinfection claim of 5 min at 35 °C.	successfully survived at −20 to 44 °C; inactivated by moist heat at 70 °C for 30 min and dry heat (160–170 °C for 1–2 h)	[80]
	Fabrics	25 days			
	Paper	6 days			
*Escherichia coli*	Glass	1–≥14 days	Susceptible to many disinfectants—1% sodium hypochlorite, 70% ethanol, phenolics, glutaraldehyde, iodines, formaldehyde	Heat sensitive, inactivated by moist heat (121 °C for at least 15 min) and dry heat (160–170 °C for at least 1 h) Dielectric-barrier discharges (DBDs) plasma	[77,78,81,82,83,84]
	Steel	14–>60 days			
	Fabrics	4 h–>8 weeks			
	Plastics surfaces	24 h–>300 days			
	Flooring materials	1 h–>8 weeks			
*Enterobacter sakazakii*	stainless steel	1–24 days	Susceptible to 70–80% ethanol, 1% sodium hypochlorite, formaldehyde, glutaraldehyde, hydrogen peroxide, iodines, peracetic acid, and quaternary ammonium compounds	Inactivated by pulsed electric fields and high hydrostatic pressure; can persist longer at higher relative humidity and low temperature	[12,85]
*Enterobacter cloacae*	stainless steel	1–24 days	Susceptible to 70–80% ethanol, 1% sodium hypochlorite, formaldehyde, glutaraldehyde, hydrogen peroxide, iodines, peracetic acid, and quaternary ammonium compounds	Can persist longer at higher relative humidity and low temperature; inactivated by moist heat (121 °C for 15 min–30 min) and dry heat (160–170 °C for 1–2 h)	[12,86]
*Pseudomonas aeruginosa*	fabrics	1 h–>8 weeks	Susceptibility has been shown for 1% sodium hypochlorite, 70% ethanol, 2% glutaraldehyde, and formaldehyde	Inactivation and sterilization by moist heat at 121 °C for 15 min or longer, dry heat at 170–250 °C or higher for 30 min or more; can persist longer at higher relative humidity and low temperature	[77,78,82,87,88]
	Plastics surfaces	9 h–10 days			
	Flooring materials	1 h–>8 weeks			
	Stainless steel	5 days			
*Staphylococcus aureus* (MRSA)	Glass	15–25 days	Susceptible to 70% ethanol, clorhexidine, 1% sodium hypochlorite, 2% glutaraldehyde, 0.25% benzalkonium chloride, and formaldehyde	Can persist longer at low humidity; can be inactivated by dry heat (160–170 °C for 1 h) but not to moist heat treatment	[77,78,82,89,90]
	fabrics	1–>70 days			
	Plastics surfaces	21 days–>3 years			
	Flooring materials	>4 h–8 weeks			
	Stainless steel	6 h–>6 weeks			
	polyethylene	90–1097 days			
*Staphylococcus aureus* (VRSA)	fabrics	1–2 weeks	Susceptible to 70% ethanol, clorhexidine, 1% sodium hypochlorite, 2% glutaraldehyde, 0.25% benzalkonium chloride, and formaldehyde	Can persist longer at low humidity, grow in a pH of 4.2 to 9.3 and in salt concentrations of up to 15%; can be inactivated by dry heat (160–170 °C for 1 h) but not to moist heat treatment	[91,92]
	polyethylene	>90 days			
	countertop	2-month			
*Enterococcus* sp.	fabrics	5 to 7 days	Susceptible to 70% isopropyl alcohol, 70% ethanol, 5.25% sodium hypochlorite, phenolic and quaternary ammonia compounds, and glutaraldehyde. Resistant to 3% hydrogen peroxide	Enterococci are killed by temperatures in excess of 80 °C	[91,93]
	Plastics surfaces	1 day			
	polyethylene	5 days–2 months			

## Data Availability

Not applicable.
